# Biosensors for Monitoring of Biologically Relevant Molecules

**DOI:** 10.3390/bios13070738

**Published:** 2023-07-17

**Authors:** Paulo A. Raymundo-Pereira

**Affiliations:** São Carlos Institute of Physics, University of São Paulo (USP), São Carlos 13566-590, Brazil; pauloaugustoraymundopereira@gmail.com

Since the creation of the glucose enzyme sensor in the early 1960s by Clark and Lyons [1], biosensors have entered a new era in the detection and tracking of biologically relevant molecules. The demand for biosensors for the management of several diseases increased tremendously, mainly after the COVID-19 pandemic caused by the SARS-CoV-2 coronavirus [2]. Biodevices can be used to detect many targets in different biofluids, including sweat, saliva, saliva, and interstitial fluid (ISF), instead of blood, and can be integrated with a portable analyzer for on-site and decentralized analysis [3]. Bioanalytical devices hold considerable promise for real-time continuous, rapid, and long-term monitoring of biologically relevant targets in bodily biofluids, the environment, pharmaceuticals, agriculture, and food safety with high sensitivity, stability, and accuracy.

Important analytical bioinformation can continuously be accessed in real time for biomedical, nutritional, therapeutic, healthcare, wellness, and disease-management applications with a new generation of advanced biosensors. The biosensing can be conducted with small molecules, including dopamine and uric acid serving as bioindicators of vital physiological functions in the complex matrix, helped by electrochemical techniques [4,5]. The colorimetric assay is also able to detect small molecules such as cortisol, known as the stress hormone, in the femtomolar range using nanomaterials (gold nanoparticles) conjugated with aptamer and antibodies to achieve the high sensitivity and specificity highly desired in biosensing analysis [6]. Complex liquid samples can be contaminated with Gram-negative and Gram-positive bacteria, which can be screened and rapidly identified with a detection platform in a fluorescent approach [7]. Fluorescence bioassays also can be applied in nucleic acid (DNA and RNA) systems in which they are important biomarkers for the prognosis and diagnosis of major diseases [8] caused by viral infections [2]. Between existent RNA, miRNA is a type of endogenous small molecule non-coding RNA with a length of about 20–24 nucleotides responsible for the synthesis of proteins, and its levels are extremely low in bodily fluids [8]. A genome-wide gene expression biosensor chip for gene expression sensing was used to examine the effects of type-I, -II, and -III IFN stimulation on the epithelial expression profiles of primary organotypic 3D air–liquid interface airway cultures [9].

Relevant biochemical markers in breath have been associated with many diseases, including cystic fibrosis, diabetes, heart failure, and lung cancer [10], making breath-reliant measurements a powerful diagnostic tool [11]. Due to the easy access to natural exhalation, sample collection is much simpler than for blood samples, and breath measurements are emerging as a promising non-invasive disease-screening tool [11]. The commercialization of breath biosensors is not easy due to the low levels of volatile organic compounds (VOCs) and interferents that influence the exhaled breath-recorded response [11]. Yet, there is a tremendous demand for feasible, fast, sensitive, robust, reliable, interference-free, and non-invasive methodologies to continuously track biologically relevant targets in breath-based analysis.
